# Enantioselective Pharmacokinetics of α-Lipoic Acid in Rats

**DOI:** 10.3390/ijms160922781

**Published:** 2015-09-21

**Authors:** Ryota Uchida, Hinako Okamoto, Naoko Ikuta, Keiji Terao, Takashi Hirota

**Affiliations:** 1 Department of Biopharmaceutics, Faculty of Pharmaceutical Science, Tokyo University of Science, 2641 Yamazaki, Noda-shi, Chiba 278-8510, Japan; E-Mail: j3b13702@ed.tus.ac.jp; 2 CycloChem Bio Co., Ltd., KIBC654R 5-5-2 Minatojima-minamimachi, Chuo-ku, Kobe 650-0047, Japan; E-Mails: hinako.okamoto@cyclochem.com (H.O.); keiji.terao@cyclochem.com (K.T.); 3 Graduate School of Medicine, Kobe University, 7-5-2 Kusunoki-cho, Chuo-ku, Kobe 650-0017, Japan; E-Mail: naoko.ikuta@people.kobe-u.ac.jp

**Keywords:** α-lipoic acid, pharmacokinetics, enantioselective, gastrointestinal availability, hepatic availability, clearance, rat

## Abstract

α-Lipoic acid (LA) is widely used for nutritional supplements as a racemic mixture, even though the R enantiomer is biologically active. After oral administration of the racemic mixture (*R*-α-lipoic acid (RLA) and *S*-α-lipoic acid (SLA) mixed at the ratio of 50:50) to rats, RLA showed higher plasma concentration than SLA, and its area under the plasma concentration-time curve from time zero to the last (*AUC*) was significantly about 1.26 times higher than that of SLA. However, after intravenous administration of the racemic mixture, the pharmacokinetic profiles, initial concentration (*C*_0_), *AUC*, and half-life (*T*_1/2_) of the enantiomers were not significantly different. After oral and intraduodenal administration of the racemic mixture to pyrolus-ligated rats, the *AUC*s of RLA were significantly about 1.24 and 1.32 times higher than that of SLA, respectively. In addition, after intraportal administration the *AUC* of RLA was significantly 1.16 times higher than that of SLA. In conclusion, the enantioselective pharmacokinetics of LA in rats arose from the fraction absorbed multiplied by gastrointestinal availability (*F_a_F_g_*) and hepatic availability (*F_h_*), and not from the total clearance.

## 1. Introduction

**Figure 1:**
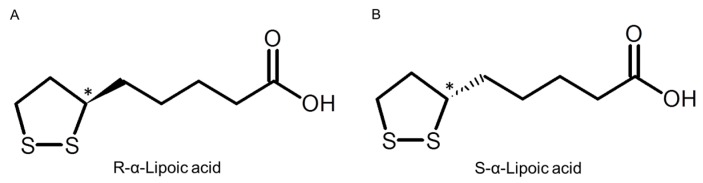
Structure of *R*-α-lipoic acid (**A**) and *S*-α-lipoic acid (**B**). Chiral center shown with asterisk (*).

In the 1930s, α-lipoic acid (LA; 5-(1,2-dithiolan-3-yl) pentanoic acid) was found to be a growth factor of bacteria [1] and was first isolated from the bovine liver in 1950 [2]. In the 1960s, LA had already started to be used for patients with liver cirrhosis [3] or mushroom poisoning [4]. In addition, a number of studies have addressed the character and efficacy of LA, and those results were reviewed by several researchers [5,6,7]. LA has been attracting a great deal of attention because of its multiple functions. LA has two sulfur atoms, one each at the C6 and C8 carbons, in its molecule. They are connected by a disulfide bond, and because the C6 carbon is chiral, LA exists as two enantiomers (*R*- and *S*-forms of LA, Figure 1).

*R*-α-lipoic acid (RLA) is biosynthesized from octanoic acid in mitochondria [8,9,10,11]. It is a natural form of LA [12], and works as a cofactor of various mitochondrial respiratory chain enzymes such as pyruvate, α-ketoglutarate, and branched-chain α-ketoacid dehydrogenases [13]. Both forms of LA seem to have different potencies. The *R*-form is more potent than the *S*-form in its ability to stimulate glucose uptake in L6 myotubes, as well as to increase insulin-stimulated glucose uptake in obese Zucker rats [14]. Endogenously-synthesized LA is covalently bound to specific proteins, which function as cofactors for mitochondrial dehydrogenase enzyme complexes [15]. In addition, to the physiological functions of protein bound LA, there is an increasing scientific and medical interest in potential therapeutic uses of pharmacological doses of free LA [16]. LA’s antioxidant properties consist of the following: (1) its capacity to scavenge reactive oxygen species (ROS) directly; (2) its ability to regenerate endogenous antioxidants, such as glutathione and vitamins E and C; and (3) its metal-chelating activity, resulting in reduced ROS production. Moreover, LA plays a pivotal role as antioxidant and metabolic component of some enzymatic complexes involved in glucose metabolism of different cell types [17]. Due largely to its antioxidant properties, LA has recently been reported to afford protection against oxidative injury in various disease processes, including neurodegenerative disorders [7]. Streeper *et al.* [18] suggested that RLA enhances insulin-stimulated glucose transport and glucose metabolism in insulin-resistant rat skeletal muscle more than SLA. Hagen *et al.* [19] suggested that RLA-supplemented aged rats had improved mitochondrial function. On the other hand, *S*-α-lipoic acid (SLA) was reported not to function as a cofactor of respiratory chain enzymes [20,21]. Moreover, Gal reported that SLA was more lethal than RLA in thiamine-deficient rats [22]. Wessel *et al.* [23] also reported that SLA caused higher mortality in streptozotocin-induced diabetic rats compared to RLA. Thus, RLA would be preferred to racemic LA (*rac*-LA) as a drug or nutritional supplement. However, because of the lower melting point of RLA (46–49 °C) than that of *rac*-LA (60–62 °C), it is difficult and costly to press into tablets. Hence, most of the LA formulations are supplied as its racemic form in consideration of its stability and ease of manufacturing.

On the other hand, several reports indeed demonstrated enantioselective pharmacokinetics of LA. Hermann *et al.* and Niebch *et al.* [24,25] suggested SLA was cleared more rapidly than RLA after infusion of *rac*-LA to humans. In addition, several groups reported the enantioselective absorption of LA after oral administration of *rac*-LA [24,26,27,28,29]. However, little is known about the mechanisms of enantioselective pharmacokinetics in detail.

In the present study, we aimed to clarify the mechanism of enantioselective pharmacokinetics of LA. Thus, we compared the systemic exposures after administration of the racemic mixture to rats by several routes and the stability of both enantiomers in different pH solutions based on the gastrointestinal conditions.

## 2. Results

### 2.1. Chiral Separation of LA (α-Lipoic Acid)

**Figure 2:**
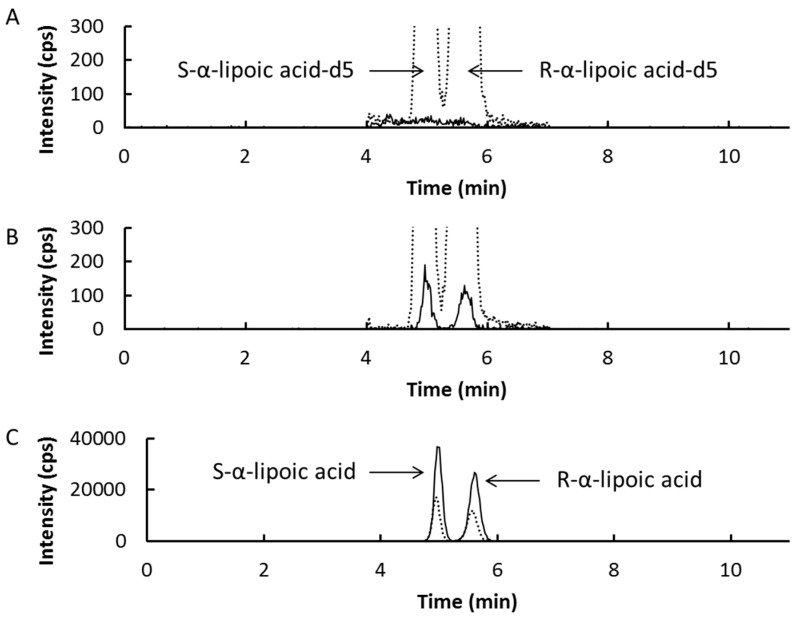
Representative chromatograms for α-lipoic acid chiral separation in plasma. Chromatograms shown with solid and dotted line are for α-lipoic acid and α-lipoic acid-d5 (internal standard), respectively. Blank plasma (**A**); blank plasma spiked with α-lipoic acid (5 ng of each enantiomer/mL, **B**); and blank plasma spiked with α-lipoic acid (1250 ng of each enantiomer/mL, **C**).

Typical chromatograms for the analysis of SLA and RLA are shown in Figure 2. The peaks with retention times of 4.95 and 5.56 min were assigned to SLA and RLA, respectively. The peak resolution calculated from the chromatograms was over 1.5. Meanwhile, the peaks with retention times of 4.92 and 5.53 min were assigned to SLA-d5 and RLA-d5 (internal standards), respectively, and its value of resolution was also over 1.5. The lower limit of quantification for each enantiomer was 5 ng/mL (Figure 2B). The range of the calibration curve was 5–1250 ng/mL, and the chromatogram of a sample at the upper limit is shown in Figure 2C. The linearity of each calibration curve was excellent (*r* > 0.999). No ghost peaks were observed (Figure 2A).

### 2.2. Stability in Different pH

The stability test of each enantiomer was performed in various pH solutions. After adding the racemic mixture to the solutions at a concentration of 10 mg LA/mL, each residual concentration in the solution was measured for 60 min. No significant difference in residual ratio was observed between the enantiomers in any of the solutions (Table 1). The solutions at pH of 1.2 and 3 were simulated gastric fluid of humans and rats [30,31,32], respectively, and that at pH of 6.8 was simulated small intestinal fluid.

**Table 1:** The stability of α-lipoic acid in various pH solutions.

Time (min)	Residual Rate (%)					
	pH 1.2		pH 3.0		pH 6.8	
	RLA	SLA	RLA	SLA	RLA	SLA
0	100	100	100	100	100	100
1	22.8 ± 3.7	22.6 ± 3.7	95.5 ± 1.0	95.4 ± 0.4	100.2 ± 2.1	100.0 ± 1.9
5	17.4 ± 2.9	17.4 ± 2.8	98.4 ± 2.0	97.8 ± 0.9	99.5 ± 0.4	99.4 ± 0.3
15	14.5 ± 3.5	14.3 ± 3.4	97.2 ± 0.7	98.1 ± 1.1	101.7 ± 1.8	101.4 ± 1.8
30	12.7 ± 1.2	12.7 ± 1.2	99.1 ± 3.2	99.2 ± 3.4	98.9 ± 1.3	99.2 ± 1.4
60	11.8 ± 0.7	11.7 ± 0.9	99.9 ± 1.4	99.7 ± 1.2	99.6 ± 1.1	99.8 ± 0.9

Residual rate are shown as mean ± standard deviation (*n* = 3). RLA, *R*-α-lipoic acid; SLA, *S*-α-lipoic acid. Statistical analysis was performed by using the paired-*t* test at each time point of each pH condition.

### 2.3. Pharmacokinetic Profiles

#### 2.3.1. Oral and Intravenous Administration

**Figure 3:**
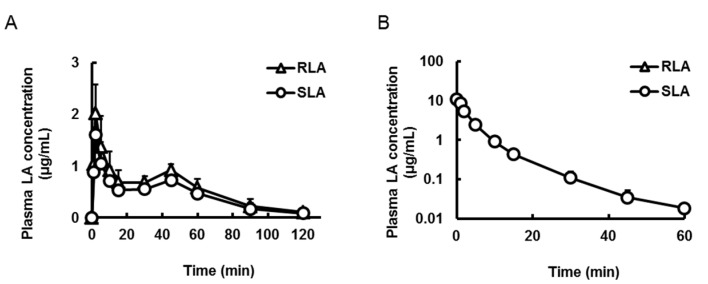
Plasma concentration-time profiles of α-lipoic acid after oral (**A**) and intravenous (**B**) administration of the racemic mixture. Data are shown as mean ± standard deviation (*n* = 4).

Plasma concentrations of LA were measured after oral administration of the racemic mixture (20 mg LA/kg, 2 mL/kg, Figure 3A) or intravenous administration (5 mg LA/kg, 1 mL/kg, Figure 3B) of the racemic mixture to rats. Although the time to peak (*T*_max_) was not significantly different between the enantiomers, the maximum plasma concentration (*C*_max_) and area under the plasma concentration *versus* time curve from time zero to the last (*AUC*) of RLA after oral administration were significantly about 1.26 times higher than those of SLA (*p* < 0.01, Figure 3A, Table 2). On the other hand, after intravenous administration, the plasma concentration profiles and their pharmacokinetic parameters the initial plasma concentration (*C*_0_), half-life (*T*_1/2_) and *AUC* were not significant different between the enantiomers (Figure 3B, Table 2).

**Table 2:** Pharmacokinetic parameters of α-lipoic acid after oral and intravenous administration of the racemic mixture to rats.

Pharmacokinetic Parameters	Oral		Intravenous	
	RLA	SLA	RLA	SLA
*C*_max_ or *C*_0_ (µg/mL)	2.0 ± 0.6	1.6 ± 0.4 *	11.4 ± 3.6	10.9 ± 3.2
*T*_max_ (min)	2.0 ± 0.0	2.0 ± 0.0	not determined	not determined
*T*_1/2_ (min)	26.7 ± 7.1	26.9 ± 7.1	10.9 ± 1.1	12.2 ± 2.3
*AUC* (µg·min/mL)	67.7 ± 6.8	53.8 ± 5.2 *	48.2 ± 3.4	46.0 ± 2.3

Pharmacokinetic parameters are shown as mean ± deviation standard (*n* = 4). RLA, *R*-α-lipoic acid; SLA, *S*-α-lipoic acid; *C*_max_, maximum plasma concentration; *C*_0_, initial concentration; *T*_max_, time of maximum plasma concentration; *T*_1/2_, half-life; *AUC*, area under the plasma concentration *versus* time curve from time 0 to the last; *, probability (*p*) <0.01 compared with RLA. Statistical analysis was performed by using the paired-*t* test.

#### 2.3.2. Absorption from Stomach and Small Intestine

**Figure 4:**
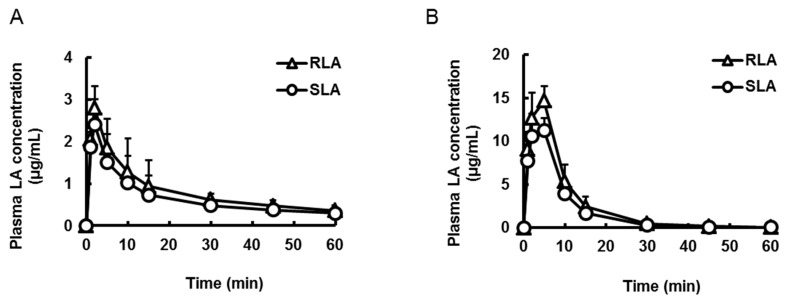
Plasma concentration-time profiles of α-lipoic acid after oral (**A**) and intraduodenal (**B**) administration of the racemic mixture to pylorus ligated rats. Data are shown as mean ± standard deviation (*n* = 4).

Plasma LA concentrations were measured after oral and intraduodenal administration of the racemic mixture (20 mg LA/kg, 2 mL/kg, Figure 4) to rats with pylorus ligation. In the case of this study, the pharmacokinetics profiles of oral administration were represented as the absorption from the stomach, because the stomach was completely separated from the intestine. As was the case with the oral administration mentioned above, although the *T*_max_ was the same between the two enantiomers, the *C*_max_ and *AUC* of RLA after oral administration were significantly about 1.16 and 1.24 times higher than those of SLA, respectively (*p* < 0.01, Figure 4A, Table 3). On the other hand, after intraduodenal administration, *T*_max_ was not significantly different between the enantiomers. Furthermore, the *C*_max_ and *AUC* of RLA after intraduodenal administration were significantly about 1.28 and 1.32 times higher than SLA, respectively (*p* < 0.01, Figure 4B, Table 3). However, the *C*_max_ and *AUC* after intraduodenal administration were several times higher than those after oral administration.

**Table 3:** Pharmacokinetic parameters of α-lipoic acid after oral and intraduodenal administration of the racemic mixture to pylorus ligated rats.

Pharmacokinetic Parameters	Oral		Intraduodenal	
	RLA	SLA	RLA	SLA
*C*_max_ (µg/mL)	2.8 ± 0.5	2.4 ± 0.6 *	14.7 ± 1.7	11.5 ± 1.7 *
*T*_max_ (min)	2.0 ± 0.0	2.0 ± 0.0	5.0 ± 0.0	4.3 ± 1.5
*AUC* (µg·min/mL)	48.1 ± 15.6	38.8 ± 13.2 *	154.2 ± 11.3	116.5 ± 4.4 *

Pharmacokinetic parameters are shown as mean ± standard deviation (*n* = 4). RLA, *R*-α-lipoic acid; SLA, *S*-α-lipoic acid; *C*_max_, maximum plasma concentration; *T*_max_, time of maximum plasma concentration; *AUC*, area under the plasma concentration *versus* time curve from time 0 to the last; *, probability (*p*) <0.01 compared with RLA. Statistical analysis was performed by using the paired-*t* test.

#### 2.3.3. Hepatic Availability

**Figure 5:**
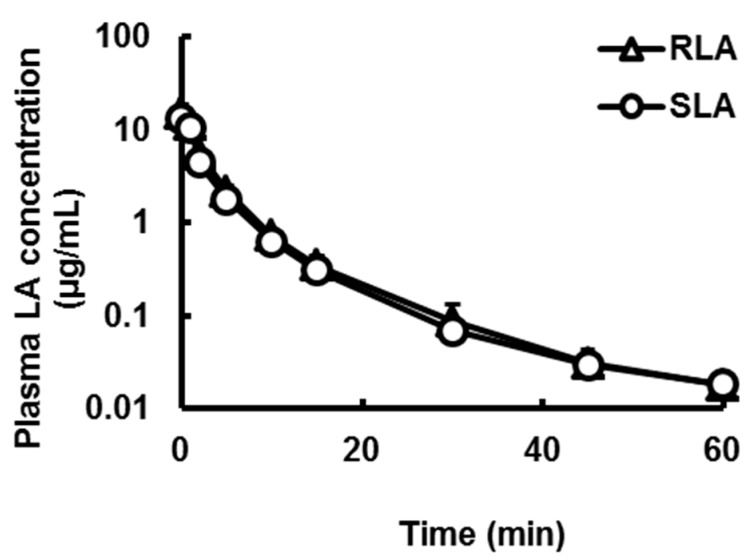
Plasma concentration-time profiles of α-lipoic acid after intraportal administration of the racemic mixture of LA to rats. Data are shown as mean ± standard deviation (*n* = 4).

To clarify the enantioselectivity in hepatic availability, rats were administered the racemic mixture of LA via the portal vein (5 mg LA/kg, 1 mL/kg) to rats. The plasma LA concentrations are shown in Figure 5. The *AUC* of RLA was significantly about 1.16 times higher than that of SLA (Figure 5, Table 4).

**Table 4:** Pharmacokinetic parameters of α-lipoic acid after intraportal administration of the racemic mixture of LA to rats.

Pharmacokinetic Parameters	RLA	SLA
*C*_0_ (µg/mL)	14.7 ± 3.9	13.2 ± 2.7
*T*_1/2_ (min)	13.9 ± 0.6	15.7 ± 1.0
*AUC* (µg·min/mL)	47.5 ± 6.1	41.0 ± 5.1 *

Pharmacokinetic parameters are shown as mean ± standard deviation (*n* = 4). RLA, *R*-α-lipoic acid; SLA, *S*-α-lipoic acid; *C*_0_, initial plasma concentration; *T*_1/2_, half-life; *AUC*, area under the plasma concentration *versus* time curve (from initial to last points); *, probability (*p*) <0.01 compared with RLA. Statistical analysis was performed by the paired-*t* test.

## 3. Discussion

In the drug development of a chiral molecule, when one of them is a eutomer (pharmacologically effective) and the other is a distomer (biologically inactive or toxic), and the enantiomers are not easily isomerized in the body, the eutomer should be provided as much as possible (FDA guideline in 1992, [33]). However, in the case of many nutritional supplements, racemic mixtures are still provided without sufficient verification of each enantiomer because the asymmetric synthesis and chiral separation are costly. Most LA formulations for supplements and drugs in the market are also racemic mixtures. Maddux *et al.* [34] reported each enantiomer was equally effective at protecting against oxidative stress-induced insulin resistance. On the other hand, Streeper *et al.* and Hagen *et al.* [18,19] reported RLA was effective in several symptoms. Indeed, the enantioselective pharmacological effects of LA might be unclear. Nevertheless, optically pure RLA was recommended in the view of safety since SLA was reported to be the distomer [22,23]. Furthermore, although several researchers suggested RLA was superior to SLA with respect to the enantioselective pharmacokinetics [24,25,26,27,28,29], the detailed mechanisms have not been revealed so far. In the present study, we examined the causes of the enantioselective pharmacokinetics.

In advance of the pharmacokinetic experiments, an assay method of LA enantiomers was developed in order to determine plasma concentrations efficiently. Previously, although the separation method was reported by Niebch *et al.* [25], it was complicated with derivatization steps and was time-consuming. Hence, we developed a simple and rapid method with reference to Kobayashi *et al.* [35] as described in the Result section. It was appropriate for the present pharmacokinetics study.

After oral administration of the racemic mixture of LA to rats, although the values of *T*_max_ were not different, the *C*_max_ and *AUC* of RLA were 1.26 times higher than those of SLA (Figure 3A and Table 2). Gleiter *et al.* [26] also reported the enantioselective pharmacokinetic profiles of LA after oral administration of a racemic mixture in humans. According to their and our results, it was found that the enantioselective pharmacokinetics in humans and rats tended to be qualitatively similar.

To clarify the mechanism of the enantioselective pharmacokinetics, the racemic mixture was administered intravenously to rats. The plasma concentration profiles and their pharmacokinetic parameters were not different between the enantiomers (Figure 3B and Table 2). Hence there was no difference in clearance between RLA and SLA. This result differed from the report of Hermann *et al.* and Niebch *et al.* [24,25], which suggested that the clearance of SLA was higher than that of RLA in humans. One of the causes of this discrepancy might be just a species difference, because the metabolic pathway of LA was proposed to be different between rats and humans [36]. From our results, at least in rats, we considered that the enantioselective pharmacokinetics after oral administration arose from the selectivity in a transporting process to the systemic circulation, probably that of absorption, or the stability in the gastrointestinal tract, and not from a difference of total clearance. Meanwhile the plasma T_1/2_ in the present study with rats was shorter than that in human studies [24,27,28]. This result was consistent with the common brief that the smaller animals were, the higher clearance they had [37].

*In vitro* stability of LA was evaluated against various pH conditions which simulated the gastrointestinal fluid. The pKa of LA is 4.7 [38], and Ikuta *et al.* [39] reported that RLA is aggregated under acidic conditions (pH 1.2). However, no difference in residual ratio was observed between RLA and SLA at pH 3.0, which was reported to be the gastric pH of fasted rats [30,31] or at pH 6.8 (Table 1). Even at pH 1.2 in simulated gastric juice, no difference was observed between the enantiomers even though some of the samples were precipitated just after mixing (Table 1). These results suggested that the cause of enantioselective pharmacokinetics did not occur before absorption. In this regard, however, it is difficult to definitively conclude that it happens after absorption. RLA and SLA were reported to have different modes of binding to proteins [40] and, thus, further experiments should be conducted *in vitro* in the appropriate conditions not only of pH but also the presence of some digestive enzymes and proteins to simulate actual gastrointestinal fluid.

Then, we examined where the enantioselective absorption occurred in the gastrointestinal tract. We previously reported that RLA was rapidly absorbed from the stomach to some extent [41]. Based on this, we evaluated the absorption separately from the stomach and small intestine after administration of the racemic mixture to pylorus ligated rats. The *AUC* of RLA after oral and intraduodenal administration was about 1.24 and 1.32 times higher than that of SLA, respectively (Figure 3 and Table 3), even though the *C*_max_ and *AUC* of each enantiomer following the small intestinal route were several times higher than those following the gastric route. These results showed that the main contribution to the enantioselective pharmacokinetics of LA arises from absorption from the small intestine.

In general, oral bioavailability (*F*) is defined as a product of the fraction absorbed (*F_a_*), gastrointestinal availability (*F_g_*), and hepatic availability (*F_h_*). Thus, intraportal administration of the racemic mixture was performed to clarify whether the fraction absorbed multiplied by gastrointestinal availability (*F_a_F_g_*) or *F_h_* contributed to the enantioselective absorption observed. The *AUC* of RLA was significantly higher than that of SLA. However, the difference was smaller than the difference in *AUC* ratio of RLA/SLA after oral administration (1.26 times). This result suggested that the enantioselectivity arose from the difference not only of *F_h_* but also of the *F_a_F_g_* in the transfer from the intestinal tract to systemic circulation. Takaishi *et al.* clearly indicated that the absorption of LA was mediated by transporters such as a monocarboxylate transporter and a sodium-dependent multivitamin transporter [42]. However, it is unclear whether the transporters contribute to the enantioselective transport because they did not assay individual enantiomers. Hence, future study using cultured cell lines will be needed to clarify whether this transport is enantioselective.

Meanwhile, the degree of enantioselectivity in rats observed in this study was not so large, that is, the RLA/SLA ratio in *AUC* is only 1.25. Gleiter *et al.* [26], reported that the difference was about 1.85 times in humans. As mentioned before, Hermann *et al.* and Niebch *et al.* indicated the clearance of SLA was faster than that of RLA in humans [24,25]. Thus, the enantioselective pharmacokinetics in humans might be caused by the differences not only in the transfer from the intestinal tract to systemic circulation but also after the transfer to systemic circulation.

## 4. Experimental Section

### 4.1. Chemical and Reagents

RLA-Na (purity > 98.0%) and SLA-Na (purity > 85.0%) were purchased from Changshu Fushilai Medicine and Chemical Co., Ltd. (Changshu, China). *Rac*-LA (purity > 98.0%) was purchased from Sigma-Aldrich Production GmbH (Buchs, Switzerland). *Rac*-LA-d5 (purity > 98.0%) was purchased from Toronto Research Chemicals Inc. (Toronto, ON, Canada). All other chemicals and reagents were commercially available and of analytical grade or higher.

### 4.2. Animals

Male Sprague-Dawley (SD) rats were obtained from Japan SLC Inc. (Hamamatsu, Japan), and used at the age of eight weeks (230–270 g) after at least one week of acclimatization. All rats were housed in a temperature—(23 ± 1 °C) and humidity—(55% ± 5%) controlled room with 12 h light/dark cycle. Water and food (Labo MR Stock, Nosan Corporation, Yokohama, Japan) were available *ad libitum* throughout the study except as described below. The rats were fasted for at least 12 h before drug administration and drugs were administered under isoflurane anesthesia regardless of the administration route. After the experiments, the rats were killed by exsanguination also under anesthesia. All rats were handled in accordance with the institutional and national guidelines for the care and use of laboratory animals.

### 4.3. In Vitro Stability Test

The simulated gastric and intestinal fluids were prepared at pH 1.2, 3.0 and 6.8. Five hundred µL of the simulated gastric fluid or intestinal fluid was added into 1.5 mL tubes, and the tubes were warmed at 37 °C in water bath for 30 min. After preincubation, 500 µL of racemic LA solution (10 mg LA/mL) dissolved in 0.5% (*w/v*) carboxymethylcellulose sodium salt (CMC-Na) was added into the tubes. Aliquots of the samples (50 µL) of the mixture were withdrawn at 1, 5, 15, 30, and 60 min under 37 °C. As a reference (time 0), water warmed at 37 °C was used instead of the simulated fluids. The collected solutions were filtered with a 0.22 µm centrifugal filter device (Ultrafree^®^-MC, Millipore, Bedford, MA, USA) by centrifuging at 4000× *g* for 15 s. Then, the filtrates were serially diluted 2500-fold with water, and stored at −20 °C until the analysis. Residual ratio was calculated by following equation.

(1) $$\text{Residual ratio }\left(\text{\%}\right)=\;\frac{peak\;area\;ratio_{each\;time\;point}}{peak\;area\;ratio_{time\;0}}\times 100$$

### 4.4. Drug Administration

#### 4.4.1. Oral and Intravenous Administration

RLA-Na and SLA-Na dissolved in CMC-Na were mixed at the ratio of 50:50 (racemic mixture), and this racemic mixture was orally administered to rats (20 mg LA/kg, 2 mL/kg, *n* = 4) by a feeding tube. The dose was determined by reference to clinical studies [43]. The racemic mixture dissolved in saline was intravenously administered to rats (5 mg LA/kg, 1 mL/kg, *n* = 4) via the caudal vein.

#### 4.4.2. Oral Administration under Pylorus Ligation

The procedure of surgery and administration was performed as previously reported [41]. Briefly, under isoflurane anesthesia, the abdomen was opened, the pylorus was slightly lifted and ligated with cotton thread, and then the incision was closed immediately with suture and an adhesive was applied. After surgery, racemic mixture was orally administered to the rats (20 mg LA/kg, 2 mL/kg, *n* = 4). The absorption from the stomach was evaluated based on the results of this experiment.

#### 4.4.3. Intraduodenal Administration

Just before pylorus ligation in the course of the operation described above, racemic mixture was injected from a syringe with a 22-gauge needle into the duodenum of the rats through the gastric corpus (20 mg LA/kg, 2 mL/kg, *n* = 4). Immediately after the injection, the pylorus was tightly ligated with suture to prevent reflux of the compound back into the stomach. The incision was closed with suture and adhesive was applied.

#### 4.4.4. Intraportal Administration

Under isoflurane anesthesia, the abdomen was opened and racemic mixture dissolved in saline was injected from a syringe with a 30-gauge needle into the hepatic portal vein (5 mg LA/kg, 1 mL/kg, *n* = 4). Immediately after the injection, the puncture was covered with adhesive and the incision was closed with suture and adhesive was applied.

### 4.5. Blood Collection

Blood was withdrawn from the external jugular vein using heparinized syringes under isoflurane anesthesia at 0 (predose), 1, 2, 5, 10, 15, 30, 45, 60, 90, and 120 min or at the same time points until 60 min after drug administration. The collected blood was centrifuged at 3000× *g* and 4 °C for 10 min to obtain plasma. Plasma was stored at −20 °C until the analysis.

### 4.6. Determination of LA Concentration by LC-MS/MS

The LC-MS/MS system consisted of API 3200™ (AB SCIEX, Framingham, MA, USA) interfaced with a Shimadzu Prominence HPLC system (Shimadzu, Kyoto, Japan). The HPLC system consisted of a LC-20AD binary pump, DGU-20A3 degasser, SIL-20A autosampler, CTO-20A column oven, and CBM-20A system controller. The measurement was performed using the method of Kobayashi *et al.* [35] with modification. Briefly, 200 µL of acetonitrile containing 0.1% (*v*/*v*) formic acid and 200 ng/mL of *rac*-LA-d5 as an internal standard was added to a 50 µL sample. After mixing, the sample was centrifuged at 10,800× *g* and 4 °C for 10 min. Ten µL of the supernatant was applied onto the LC-MS/MS system. The HPLC was fitted with a CHIRALPAK AD-RH column (5 µm, 2.1 × 150 mm, Daicel, Osaka, Japan), and chromatography was performed using a gradient elution program at a flow rate of 0.3 mL/min. The column temperature was maintained at 30 °C. The mobile phase consisted of 0.1% (*v*/*v*) formic acid/water (A) and 0.1% (*v*/*v*) formic acid/methanol (B). The gradient program was as follows: start the ratio of A/B at 60/40, increase B linearly from 40% to 95% between *t* = 0 and 1.0 min, then hold the ratio of A/B at 5/95 until *t* = 6.0 min, decrease B linearly from 95% to 40% between *t* = 6.0 and 6.1 min, then hold the ratio of A/B at 60/40 until *t* = 11.0 min. The analytes and internal standard from the column were detected by the negative ion mode, and analyzed by multiple reaction monitoring mode of the transitions *m*/*z* 205.0 to 170.8 for *rac*-LA and *m*/*z* 210.0 to 173.8 for *rac*-LA-d5.

### 4.7. Pharmacokinetics Analysis

The *C*_max_ and *T*_max_ were obtained directly from the individual plasma concentration-time profiles. The *C*_0_ and *T*_1/2_ were calculated using the macro program MOMENT (EXCEL) [44]. The first and last three points of the logarithmic plasma concentration were used for linear regression of *C*_0_ and *T*_1/2_, respectively. The *AUC* of LA was estimated using the trapezoidal rule.

### 4.8. Statistical Analysis

Parameters are presented as arithmetic mean ± standard deviation. The pharmacokinetic parameters were compared with paired-*t* tests for comparison of each enantiomer. Differences were considered statistically significant when *p* < 0.01.

## 5. Conclusions

We revealed the following findings about enantioselective pharmacokinetics of LA. (1) The exposure to RLA is higher than that to SLA; (2) The enantioselectivity occurred in the absorption phase, and not in the elimination phase. The *F_h_* and *F_a_* and/or *F_g_* in a transfer process from the gastrointestinal tract, mainly the small intestine, to systemic circulation are implicated in the enantioselective pharmacokinetics. According to these results and the observation that RLA is a eutomer, a formulation of the single enantiomer RLA would be more suitable for oral administration of LA than that of a racemic mixture.
